# Midwifery academic educators' experiences and knowledge on managing students' moral distress and psycho-emotional trauma during clinical placement: A qualitative study protocol

**DOI:** 10.3934/publichealth.2026038

**Published:** 2026-06-26

**Authors:** Maria Karanikola, Virginia Koutroubas, Nicos Middleton, Anne Griese, Eleni Hadjigeorgiou, Veronika Christodoulides, Ourania Kolokotroni, Julia Leinweber

**Affiliations:** 1 Department of Nursing, Cyprus University of Technology, Limassol, Cyprus; 2 Institute of Midwifery, Charité Center for Prevention, Health and Human Sciences, Charité – Universitätsmedizin Berlin, (Berlin), Germany; 3 Gostthinker GmbH, Augsburg, Germany

**Keywords:** moral distress, psycho-emotional trauma, midwifery education, clinical placement, academics, educators, students, pedagogy, ethics

## Abstract

While growing attention has been given to moral distress and work-related psycho-emotional trauma among practicing midwives, few researchers have explored how midwifery academic educators understand, identify, and manage these phenomena in students. This paper presents a study protocol on the exploration of midwifery academic educators' experiences and knowledge in managing students' moral distress and psycho-emotional trauma related to clinical placement. A qualitative research design using focus group interviews with academic educators involved in undergraduate and postgraduate midwifery education will be adopted. Participants will be purposively recruited according to predefined inclusion and exclusion criteria. Focus groups will consist of 6–8 participants. Two focus group discussions will initially be conducted in each country in the host country's native language. Following preliminary data analysis and identification of major- and subcategories, a second round of focus group confirmatory discussions will be conducted with the same participants. Data will be collected through semi-structured discussions focusing on participants' direct or indirect experiences regarding midwifery students' moral distress and trauma, pedagogical approaches academic educators' use to address these issues, strategies for identifying and managing students' distress, and practices that facilitate students' disclosure and psychological safety. Data will be analysed using the inductive content analysis approach. The study is expected to provide insight into current educational practices, perceived challenges, and gaps in academic educators' preparedness to address moral distress and psycho-emotional trauma among midwifery students. Findings are anticipated to inform the development of targeted pedagogical strategies, continuing education initiatives, and supportive interventions aimed at enhancing students' well-being and resilience during clinical placement. By highlighting academic educators' perspectives, this study seeks to contribute to the advancement of midwifery education through the promotion of psychologically safe learning environments and evidence-informed approaches to managing moral distress and maternity care-related trauma in academic and clinical training contexts.

## 1. Introduction

Moral distress in clinical practice refers to the psychological burden felt when individuals encounter ethically challenging situations and are unable to act in accordance with what they consider to be the right thing to do [1],[2]. Most of the time, moral distress is experienced when clinicians are not able to provide the care they consider patients need [1]. Emotional and psychological responses associated with moral distress include fear, guilt, conflict, frustration, anger, and feelings of violation [3]. Although the construct has been interpreted in various ways, a widely cited definition describes moral distress as the emotional, psychological, and physiological suffering that clinicians may experience when, constrained by circumstances, they participate in perceived wrongdoing, either by action or omission [4].

Several profession-specific factors may contribute to moral distress in midwifery that differ from those experienced by other healthcare providers. Midwives often feel responsible for safeguarding the interests of the birthing woman and the fetus. This dual obligation can intensify ethical tension, particularly in areas such as pregnancy termination or women's choice of birth mode, where midwives' role, responsibilities, and clinical expertise introduce additional layers of complexity [2],[5]. These challenges are compounded by conflicting values, power dynamics, and professional philosophies between midwives and medical practitioners [6]. Collectively, these factors shape a professional landscape in which midwives may experience moral distress in distinct and multifaceted ways.

Moral distress has important implications for midwives' mental health and occupational well-being and has been reported to affect up to 30% of professionals who have considered leaving their occupation [7]. Despite its recognized impact, the identification and measurement of the phenomenon remain challenging, highlighting the need for more comprehensive tools to assess and better understand its spectrum [8]. Researchers have employed Delphi methodologies involving academics and midwife educators to develop statements describing moral distress incidents in midwifery clinical education [9],[10]. However, empirical evidence on the living experiences of midwives' moral distress remains limited, and data concerning students and midwife-mentors are particularly scarce [11]. Specifically, students report experiences of moral distress when they witness care that deviates from clinical guidelines or is perceived as ineffective, inappropriate, or constituting malpractice, such as unnecessary interventions, neglect, or disregard for women's autonomy, also underscoring lack of preparation to manage moral challenges related to clinical placement [12],[13]. These situations often evoke feelings of powerlessness, frustration, and guilt [14]. Furthermore, studies highlight a lack of systematic ethics education within midwifery curricula [15],[16]. Students report relying primarily on mentors, preceptors, and role models to make sense of ethical challenges, with limited formal instruction on how to navigate them [17],[18]. This gap seems to leave learners particularly vulnerable when confronted with ethically complex clinical situations, and educators unable to identify and manage relevant phenomena [19].

Indeed, there is a notable lack of research exploring midwifery academic educators' knowledge and experiences in managing moral distress and psycho-emotional trauma in students [19]. Students' exposure to morally distressing and traumatic experiences early in training may result in severe psycho-emotional suffering, avoidance behaviors, and, in some cases, their premature withdrawal from the profession [13],[20]. These outcomes underscore the importance of early identification and structured educational support. Moreover, midwifery academic educators play a central role in preparing students for clinical placement in maternity care settings through theoretical instruction, skill development, and mentorship. Beyond practical and academic competencies, they are instrumental in supporting students' psychological preparedness and resilience during their transition into clinical practice [14]. Research shows that healthcare students and early-career professionals, including midwifery students, may encounter potentially traumatic and morally distressing experiences [4],[7],[14],[21]–[23]. These phenomena are mostly rooted in organizational culture, ethical conflicts, and systemic constraints [11],[24]. Such experiences have been associated with reduced safety of the care provided, ethical and human rights violations, diminished professional identity, and disengagement attitudes, including quiet quitting [11],[24],[25].

Despite growing awareness, moral distress and trauma are not adequately addressed within midwifery educational curricula, with meaningful attention to these phenomena emerging only in recent years [9]. Here, we present a protocol for a qualitative study to assess midwifery academic educators' experiences and knowledge on managing students' moral distress and psycho-emotional trauma related to clinical placement. The goal of this study is to examine how academic educators identify, reflect on, and pedagogically address these phenomena, facilitate students' disclosure, and initiate relevant discussions or interventions with students and teaching personnel within educational settings. This knowledge is expected to contribute to the development of safe learning environments and innovative pedagogical strategies, including blended learning approaches, to enhance educational quality, students' support, both theoretical and clinical, and mental well-being during clinical placement [26].

In the long run the findings may inform: a) Pedagogical approaches on moral distress and psycho-emotional trauma related to midwifery students' clinical placement; b) continuing education strategies on these topics; c) academics' and educators' competencies and skills for managing such issues in midwifery students [4]; d) the creation of safe spaces for reflection and dialogue; and e) the design of innovative, effective interventions to mitigate these phenomena in academia [27].

## 2. Materials and methods

### 2.1. Aim & objectives

The aim of this study is to collect qualitative data to reveal the breadth and depth of midwifery academic educators' experiences, perceptions, and knowledge on morally distressing and emotionally traumatizing maternity care-related experiences in students in Germany and in the Republic of Cyprus (RC). The major objectives are to explore midwifery academic educators': a) Personal experiences with traumatic and/or morally distressing experiences, either directly (as a clinician) or indirectly, through their contact with service users; b) knowledge on students' and midwife-mentors' traumatic & morally distressing experiences related to maternity education clinical placement; c) their pedagogical approaches to these issues; and d) their experiences and readiness on encountering/managing these phenomena.

Since there is no data regarding these issues among the Greek-Cypriot and German population, and international relevant studies are limited, a qualitative, exploratory methodology for data collection and analysis will be applied to provide a more comprehensive understanding of these phenomena. The reporting of findings will be guided by the consolidated criteria for reporting qualitative research (COREQ) checklist for qualitative studies [28].

### 2.2. Target population

In this study, the term “midwifery academic educators” refers to university-based academic staff who teach midwifery students at a higher education level. Thus, the target population comprises academics and educators who teach midwifery students at private and public universities in both countries. Clinical mentors/preceptors in placement settings are not included in the target group of this study.

### 2.3. Sampling

The following criteria will be used to select the participants of the focus group:

(a) Employment in academia as a lecturer/professor or clinical instructor/ teaching assistant with active involvement in the academic preparation of midwifery students.

(b) Excellent knowledge and understanding of the Greek/German language since the interview guide for data collection has been developed in Greek/ German.

(c) A written expression of willingness to participate in the research, informed by a completed consent form.

Exclusion criteria:

Midwife mentors whose role involves only clinical training. The professional demands, pedagogical expectations, and continuing education structures of clinical mentors employed in hospital organizations differ markedly from those of an academic faculty. Including this set of participants could introduce heterogeneity, thereby obscuring patterns specific to the academic context and limiting the validity and interpretability of the findings. Therefore, this criterion is essential for ensuring conceptual and contextual consistency within the dataset.

Participants will be selected by purposive sampling to ensure a specific composition in the group; to ensure homogeneity in terms of commonality, i.e., being academics/educators, but differentiated from each other in terms of their experiences, age, range of work experience, and disciplines to enable the expression of different or contradictory views [18],[29],[30]. Selecting a sample with experience across disciplines and research topics will ensure this delicate balance between homogeneity and diversity.

A form describing the launch of this study, in a clear and understandable way, indicating the purpose, objectives, and the methodology to be applied, will be sent to the educational institutions from which the participants will be included, i.e., the CUT, Limassol, and the Charité, Berlin, using professional contact information. Thus, eligible participants will receive an invitation email containing information about the study and instructions on how to express interest in participation. In addition, the researchers' contact details will be provided so that people wishing to participate in the focus groups can contact them.

The size of the focus groups has been set at 6–8 persons.

### 2.4. Data collection

Data will be collected through focused discussion groups and a semi-structured interview guide [18],[30]. Aiming to explore academic educators' shared experiences and perspectives regarding the management of students' moral distress, focus groups will be selected rather than individual interviews. Focus groups facilitate interaction among participants, enabling educators to reflect on and respond to each other's experiences, which can generate richer insights into common challenges, norms, and strategies within educational contexts. This interactive format is particularly valuable for examining socially situated experiences, such as teaching and mentoring practices within academic environments. In addition, the topic of the study focuses on educators' professional experiences and pedagogical approaches rather than sensitive personal information or identifiable personal data. Therefore, a focus group setting will be considered appropriate and unlikely to inhibit participants' willingness to discuss their perspectives openly [31].

Initially, there will be at least two focus group discussions in each country, conducted in the host country's mother tongue (German in Germany and Greek in Cyprus). Following data analysis and extraction of the major- and subcategories, a second set of focus group discussions with the same participants will be held. The aim of this second discussion will be to confirm and enrich the data extracted from the first group discussion. This step aims will aim to enhance the credibility of the analysis through participant validation of the researchers' interpretations. Thus, it will be shorter and more focused than the initial discussions, concentrating on validating emerging themes. The results from both focus group discussions will be translated into English. The planned number of focus groups has been informed by methodological guidance for qualitative research and the expectation that this number will enable sufficient exploration of participants' perspectives while enabling the identification of recurring themes. Nevertheless, data collection will continue until sufficient thematic depth and saturation are reached [30],[32].

Then, a third focus group discussion will take place in English with participants from both countries. The aim of this cross-national mixed-sample discussion will be to confirm and enrich the major categories and subcategories of themes addressed in each country. Each country will have at least three delegates in the mixed-sample discussion. Participants will be informed of this requirement in advance, and participation will therefore assume a sufficient level of comfort communicating in English. During the discussion, participants will also be allowed to briefly use words or expressions in their native language if needed, with clarification provided by the research team to ensure accurate understanding.

#### 2.4.1. Demographics data collection tool

Personal data will be collected via a questionnaire to record demographics (gender, age, place of residence, marital status, education, etc.) and occupational characteristics (rank, discipline/research topic, clinical experience (field/years); if relevant, experience (years) in academia.

#### 2.4.2. Empirical data collection tool - interview guide

Data will be collected with an interview guide encompassing open-ended questions developed by the research team, following an extensive literature review aligning with our objectives [7]–[10],[18],[19],[23]. The interview guide will be pilot-tested and reviewed accordingly, prior to data collection to ensure clarity, relevance, and appropriateness of the questions. Feedback from the pilot will be used to refine the guide where necessary.

The following questions will be included in the interview guide:

1. Can you describe a time during your clinical practice when you were certain about the right clinical and ethical decision to be made, but you were unable to act upon and follow it? What factors influenced how you responded in that situation? Were there any constraints or pressures that affected your actions? How did you experience that situation at the time?

2. Can you describe to us any challenging or emotionally demanding experiences you have encountered in your professional role, either directly in clinical practice or through your interactions with service users or colleagues? How did you experience or respond to these situations?

3. Have you encountered accounts of challenging professional experiences indirectly, for example through discussions with colleagues? If so, could you describe one?

4. Can you describe to us your prior knowledge on students' morally and emotionally distressing experiences related to their educational maternity-specific clinical placement? How did you get this knowledge?

5. How do you typically address students' distressing or emotionally demanding experiences that arise during educational maternity-specific clinical placement? What strategies or approaches do you use when supporting students?

6. Can you describe to us whether your own professional morally or emotionally distressing experiences influenced how you teach or support students regarding challenging situations in clinical placement? If so, in what ways?

7. Can you describe any situations in which you have supported or guided students encountering morally and emotionally distressing experiences during maternity-specific clinical placements? What aspects of the situation were challenging? How did you approach it? What did you learn from the experience? Are there any obstacles or skills you think are important in these situations?

8. What is your understanding of the experiences midwife-mentors may have in their clinical teaching roles, particularly when supporting students in morally and emotionally challenging situations?

Table 1 presents of the alignment between study objectives and interview guide.

**Table 1.** Presentation of the alignment between study objectives and interview guide.

Aim of the Study				
Revelation of the breadth and depth of midwifery academic educators' experiences, perceptions, and knowledge on morally distressing and emotionally traumatizing maternity care-related experiences in students in Germany and in the Republic of Cyprus (RC).				
Study Objectives
Midwifery academic educators' personal experiences with traumatic and/ or morally distressing experiences, either directly (as a clinician) or indirectly, through contact with service users.		Midwifery academic educators' knowledge on students' and midwife-mentors' traumatic & morally distressing experiences related to clinical placement.	Midwifery academic educators' pedagogical approaches to midwifery students.	Midwifery academic educators' experiences and readiness on encountering/managing students' morally & emotionally distressing experiences.
Interview Guide Questions
Can you describe a time during your clinical practice when you were certain about the right clinical and ethical decision to be made, but you were unable to act upon and follow it? What factors influenced how you responded in that situation? Were there any constraints or pressures that affected your actions? How did you experience that situation at the time?	Can you describe to us any challenging or emotionally demanding experiences you have encountered in your professional role, either directly in clinical practice or through your interactions with service users or colleagues? How did you experience or respond to these situations?	Can you describe to us your prior knowledge on students' morally and emotionally distressing experiences related to their educational maternity-specific clinical placement? How did you get this knowledge?	How do you typically address students' distressing or emotionally demanding experiences that arise during educational maternity-specific clinical placement? What strategies or approaches do you use in these cases?	Can you describe any situations in which you have supported or guided students encountering morally and emotionally distressing experiences during maternity-specific clinical placements? What aspects of the situation were challenging? How did you approach it? What did you learn from the experience? Are there any obstacles or skills you think are important in these situations?
Even if you did not have direct experience of such cases, did you come across them indirectly, e.g., through conversations with colleagues? How did you feel then?			Can you describe to us whether your own professional morally or emotionally distressing experiences influenced how you teach or support students regarding challenging situations in clinical placement? If so, in what ways?	
		What is your understanding of the experiences midwife-mentors may have in their clinical teaching roles, particularly when supporting students in morally and emotionally challenging situations?		

#### 2.4.3. Focus group discussions

The use of focus group discussions will enable participants to present their responses simultaneously, highlighting the most important issues as perceived and prioritized by the participants. The interaction of group members is an essential source of information for the researcher [30],[32], enriching the analysis by indicating issues related to the dynamics that may emerge during group discussions.

*Process of organizing and conducting focus group discussions*. The focus groups will be organized online, away from the work environment, or in a meeting room of the hosting university. Participants will be informed about the purpose and process of the study, security, confidentiality, and privacy regarding the data to be collected, through a written consent form and verbally.

The moderator of the groups will be responsible for welcoming participants, providing information about the topic to be discussed, explaining and ensuring that the discussion rules are respected, and facilitating the opportunity for participants to get to know one another. He or she will ensure there are no distractions and that no participant monopolizes the discussions. The facilitator will enable a ‘strong’ personality to lead the discussion to some extent to ‘draw others into it’ (leveraging the dynamics that develop in a group), but without allowing this person to impose his or her positions [30],[32]. It is noted that the moderator will not influence the discussion by projecting personal positions or by asking direct questions to ensure the validity of the research process. Furthermore, it is noted that the facilitator will not have a power/power/dependence relationship with the participants. The discussion will be recorded to ensure the accuracy of the narratives, provided permission is secured from the participants. Each discussion is expected to last approximately 1.5–2 hours.

### 2.5. Data analysis

The analysis of the data will be performed by three independent analysts, members of the research team with formal education in qualitative data analysis and extensive experience in content analysis in particular.

This process will initially involve listening to the recorded interviews and transcribing them. Listening to the recorded interviews and reading the transcripts will enable researchers to deepen their understanding of each participant's narrative and take notes on nonverbal cues, such as the tone of voice and emotional expressions. This is a crucial step in the research process that can foster a more personal connection with the participants, even when they are not present. Approximately one week after the interview, each researcher will review the transcribed interview to identify and underline the participants' perceptions and experiences regarding the research questions of the study. Specifically, they will record their comments on participants' narratives, the meaning of their experiences, their behaviors, feelings, expressions, and emotional tensions, and the constraints they faced. To achieve this, the researchers will be guided by the template presented in Table 2. This template has been developed for the purpose of this study. Prior to the next phase of the analysis, consecutive meetings will be held among all three researchers to determine the degree of agreement between the observations. If the degree of match is satisfactory, the next step of the analysis will follow. In this phase, codes, subcategories, and categories will be created to reflect common feelings and shared experiences, from which common, preliminary topics will emerge. This process will also determine the saturation phenomenon, which occurs when there are no new codes, and only repetitive codes emerge.

To ensure the rigor, trustworthiness, and transferability of the study results, the triangulation method for data analysis will be employed, as mentioned above. Specifically, the content analysis team will be comprised of the principal researcher and two more academically prepared researchers who are formally educated and experienced in qualitative data analysis. They are expected to independently analyze each focus group interview before collectively reviewing and validating the codes and categories generated by each researcher. This approach is expected to enhance transparency by integrating diverse perspectives and preventing data loss, thereby ensuring the credibility of the results. The quality of the findings and confirmation of the study's rigor and trustworthiness will be evaluated using interpretive criteria consistent with Munhall's perspective [33] (Table 3).

Through collaborative efforts, code groups will be established to reflect the participants' common interpretations, descriptions, and perceptions. For consistency in terminology, the main terms, i.e., “moral distress”, “primary trauma”, “secondary trauma”, and “work-related psycho-social risks”, will be defined, as well as constructs related to the main terms, such as moral injury, moral residue, and moral dilemma (Table 4). These codes are expected to facilitate the emergence of key categories and sub-categories. Descriptive definitions will be further developed for each category, sub-category, and emerging codes, ensuring clarity in data selection and interpretation. Initial categories and sub-categories will be formulated directly from the data, guided by the pre-established definitions. Yet, by following an inductive approach, the analysis will not be restricted by a pre-existing theoretical framework. Subsequently, levels of abstraction will be identified, enabling the development of additional codes, categories, and themes, all grounded in the same data-driven framework.

Continuous peer debriefing will be conducted throughout the coding and theme development process to support reflexivity and interpretative consistency. Moreover, as part of the analytical process, the research team will systematically review the data for contradictory or disconfirming evidence. Most importantly, as the study includes participants from Germany and Cyprus, contextual and cultural factors will be considered during analysis. Although it was not aimed to conduct a formal cross-national comparison, attention will be given to how participants' accounts from both countries may reflect their respective educational and clinical contexts. During analysis, themes will therefore be examined and compared across the two settings to identify shared experiences and potential context-specific differences while remaining sensitive to cultural variations between the two settings.

Furthermore, and for the purposes of analysis, data collected in German and Greek will be translated into English. To preserve the meaning and contextual nuances of participants' accounts, translation will be conducted carefully by bilingual members of the research team familiar with the study context. Particular attention will be given to maintaining the semantic and cultural meaning of participants' expressions, especially when discussing emotionally or ethically sensitive experiences. Where necessary, original language excerpts will be revisited during analysis to ensure that interpretations accurately reflect participants' intended meanings.

**Table 2.** The data analysis template, with an example included.

INTERVIEW X				
Interview quote	Condensed meaning	Codes/ Comments	SUB-CATEGORY	CATEGORY
*Interview guide question 1*				
Participant X: During childbirth, if we have difficulty in the course of labour, fetal or maternity distress; this is certainly a stressful situation, because there is no obstetrician- he is not in the clinic but at his home.	“[...]During childbirth […] difficulty in the course of labour […] stressful situation[...] no obstetrician [...]”	Job strain situation: -Complications of childbirth (Fetal & maternal distress)	Occupational stressors/ Complications in labor	Work-related distressing experiences
		Job strain situations: -Absence of an obstetrician ≥ psychosocial risk	Occupational psycho-social risks /\Physicians' absence & lack of compliance with work hours	

**Table 3.** Presentation of the criteria aiming to ensure the rigor and trustworthiness of the study.

Criterion	Description	How the criterion will be supported in the study
Reasonableness	The results constitute a reasonable interpretation of the phenomenon	The findings will be confirmed a) in the phase of repeated interviews, and b) during the presentation of the findings to the participants; Participants' expressions such as *“Yes, it seems logical to me,”* and *“Yes, it makes sense to me”* will be deemed as confirmatory statements. The integrity of the data analysis process will be assured by continuous meetings of those analyzing the data to determine the degree of consensus among them regarding the findings. In the event of any differences in opinion, they will engage in extensive dialogue to reach a consensus.
Resonance	The analysis of the phenomenon reverberates with participants	The analysis will be corroborated through participants' feedback during iterative interviews and presentation of findings. This will be evidenced by statements such as *“Yes, that accurately reflects my perspective.”* Moreover, through repeated meetings with each participant, the researcher will allow sufficient time to build a relationship, facilitating the sharing of experiences.
Representativeness	In-depth interpretation of the phenomenon	Aiming to ensure data saturation, the following measures will be implemented: (a) Participants from different disciplines, having adequate experience as academics/educators, both genders, and diverse family status (single/divorced/married) will be represented equally, or almost equally; b) participants will be prompted to illustrate their experiences with concrete examples, thereby fostering a comprehensive understanding of their meaning; c) interviews will be concluded only when participants indicate that they have exhausted their responses, using a closing query such as, “Is there anything further you'd like to include?”; (d) the time spent on data collection and analysis will be sufficient, considering the sample size, the complexity of the study design, and the scope of the phenomenon; and (e) all participants will engage in a follow-up interview, allowing for reflection and potential expansion or revision of their initial accounts. By conducting supplementary interviews and spending time deepening the understanding of the data during analysis and interpretation, the degree of data and theoretical saturation will be enhanced.
Relevance	The relevance of the study to the objectives of the qualitative paradigm.	The study's conclusion will align with participants' experiences and self-perceptions, as revealed in their narratives.
Recognizability	Readers recognize aspects of their experience in the interpretation	The applicability of the findings will be confirmed through the presentation of these findings to midwives, students, educators and academics from different healthcare settings and to healthcare administrators, citing expressions such as: *‘What you are describing to me, I have witnessed firsthand in my department/organization/clinical placement’*.

**Table 4.** Definitions to contextualize data analysis.

Construct	Definition	Clarifications
Moral Distress [1]	Occurs in specific situations where clinicians believe they know the morally right action but feel prevented from carrying it out due to external constraints (e.g., institutional policies, hierarchy, lack of authority).	• Episodic and situation-specific • Involves perceived constraints & power imbalance • Powerlessness is a core experience • Results in negative emotions such as guilt, frustration & shame
Moral Dilemma [3]	A situation in which two or more morally justifiable actions conflict and there may be no clearly correct choice, creating ethical uncertainty.	• No single morally correct option • Ethical principles may conflict • Arises when uncertainty about the right action exists
Moral Stress [34]	A form of stress arising from the routine operation of overstressed healthcare systems that prevents clinicians from consistently providing care aligned with professional standards and patient needs. It is produced by systemic conditions rather than specific clinical encounters	• Chronic and pervasive in healthcare systems • Often system-generated (e.g., bureaucracy, resource limitations) • Not necessarily associated with feelings of powerlessness • Directs attention to structural/system factors rather than individual decisions
Moral Injury [35]	A lasting psychological injury that results when individuals must violate their moral conscience while performing professional duties, often in broken or severely stressed systems. This violation may or may not be relevant to moral distress.	• Deep, enduring damage to moral identity or integrity • May arise from a single event or accumulation of moral distress • Associated with profound loss of trust, hope, or moral coherence • Is described as a trauma-related syndrome.
Moral Residue [36]	The enduring psychological suffering remaining after episodes of moral distress, which can accumulate over time and contribute to ongoing moral burden.	• Persistent after the triggering event • May accumulate (“crescendo effect”) • Reflects unresolved moral-related trauma associated with previously experienced situations
Work-related psychosocial risks [37]	Anything in the design or management of work that increases the risk of work-related stress and can be understood as a psychosocial hazard.	• They are part of the work-related stressors
Psycho-emotional Trauma (primary & secondary) [38],[39]	A disturbing experience resulting from any cause, resulting in significant fear, helplessness, dissociation, confusion, or other disruptive feelings intense enough to have a long-lasting negative effect on a person's attitudes, behavior, and other aspects of functioning.	• Traumatic events, mainly include- but are not limited to- those caused by human behavior and often challenge an individual's perception of the world as a just, safe, and predictable place.
Maternity care-related trauma [39],[40]	Maternity care-related trauma refers to exposure to distressing or potentially traumatic events in maternity care.	• Midwives/students may be themselves subjected to brutal behaviors e.g., verbal or physical violence, or they may be exposed to the severely distressing events of the women they care for. • Midwives/ midwifery students often have an emotional close relationship with the woman in their care. Midwives/ midwifery students are likely to be directly traumatized by witnessing the woman experiencing life-threatening situations.

## 3. Ethical considerations

The design of the study conforms to the provisions of the Declaration of Helsinki. Permission will be obtained from the National Bioethics Committee for the RC and Germany to conduct the research.

Participants will be asked to volunteer with the assurance that all information will be kept confidential. Completion of the questionnaire by participants will also constitute their voluntary consent to participate in the research. This will be clearly stated in the instructions, including that the participant's identity will be coded and cannot be disclosed or linked in any way to participation in the focus group. The data extracted from the focus groups will be stored at the Cyprus University of Technology, Department of Nursing, during the period of analysis and dissemination of the research results, and will be destroyed 5 years after publication of the results.

## 4. Discussion

A protocol for a qualitative study designed to explore academics' and midwifery educators' experiences, knowledge, and pedagogical readiness in managing midwifery students' moral distress and psycho-emotional trauma related to clinical placement was presented in this paper. The proposed study is intended to provide an initial understanding of how moral distress and trauma-related experiences are perceived, recognized, discussed, and managed in midwifery education.

The findings are expected to generate valuable insights into academics' and educators' pedagogical and clinical practice experiences, shared patterns of practice, and context-specific challenges. By focusing on two educational and clinical contexts, i.e., Greek-Cypriot and German, it is aimed to provide data to a notable gap in the national and international literature, where empirical evidence of these topics remains limited. Moreover, the inclusion of multiple contexts is expected to strengthen the transferability of findings and support future collaboration in the design and implementation of educational interventions.

An important strength of the proposed study lies in its focus on the relationship between moral distress and psycho-emotional trauma. Although research shows that midwifery and maternity care students experience moral distress [4] and traumatic responses during clinical placement [40],[41], the intersection and potential interaction of these constructs remain understudied [42],[43]. This requires further research as both phenomena have been shown to influence midwifery students' professional attitudes, coping responses, and professional identity formation [44],[45]. To the best of our knowledge, the proposed study will be among the first to explore these experiences concurrently and for the first time from the perspective of academics and educators involved in midwifery studies.

Furthermore, the persistent dissonance between the ethical values and standards promoted in academic settings and the realities of clinical practice underscores the need for greater alignment between educational ideals and real-world clinical expectations [14]. Researching academics' and educators' knowledge and pedagogical approaches in relation to students' moral distress and trauma may contribute to improved student engagement, confidence, and well-being during clinical placement [46]. Understanding how educators navigate this tension is therefore critical for strengthening educational and clinical learning environments.

Given the limited consensus in the literature on the effectiveness of educational interventions for moral distress and trauma prevention [19], a key contribution of the proposed study lies in mapping what academics and educators know, experience, and implement in practice. By identifying strategies and perceived gaps, the findings are expected to inform the development of structured, evidence-informed pedagogical interventions and continuing professional education initiatives tailored to educators' needs.

The anticipated findings may guide the design of targeted educational interventions that support educators and students. Teaching content related to emotionally challenging experiences, moral conflict, and distressing clinical realities requires careful, intentional pedagogical planning, with clearly defined goals [15]. Advancing trauma-informed and morally grounded pedagogical approaches, strengthening moral competencies and communication skills, and creating psychologically safe classroom and mentoring spaces where difficult clinical experiences can be reflected upon are potential contributions of the proposed research.

Moreover, evidence underscores the complexity of moral distress in midwifery practice. In a review, researchers identified multiple contributing factors, including societal and economic pressures, resource limitations, workplace culture, power imbalance, mistreatment of service users, defensive practices, and exposure to extreme clinical situations [11]. Addressing such multifaceted issues through education alone is challenging. However, the findings of this study may introduce additional causes or contextual nuances relevant to educational settings. This complexity further supports the need for ongoing professional strategies for midwifery educators and mentors, including blended learning approaches that emphasize reflective practice, psychological safety, and moral resilience [21].

Furthermore, the use of focus groups for data collection enables academics and educators to collectively reflect on sensitive issues, exchange perspectives, and articulate shared or divergent pedagogical assumptions [47]. Additionally, inductive content analysis enables theme categories to emerge directly from participants' language and meaning-making processes, offering a nuanced understanding of how moral distress and psycho-emotional trauma are conceptualized and addressed in educational practice [29].

Together, these methodological choices are expected to provide a rich and contextualized account of current practices and future needs.

Overall, we aim to contribute to the development of more supportive, sustainable, and ethically responsive educational environments. By providing data on tools, methods, and strategies to strengthen educators' capacity to address moral distress and psycho-emotional trauma, our findings have the potential to contribute to the improvement of midwifery students' educational experiences, clinical preparedness, and long-term professional well-being.

## 5. Limitations

As a qualitative study, the proposed research has several limitations that should be acknowledged. First, the findings will be based on self-reported data from academics and midwifery educators, which may be influenced by recall bias, social desirability, and participants' willingness to disclose sensitive experiences related to moral distress and psycho-emotional trauma or fear of revealing sensitive clinical practices. While focus groups can foster rich discussion, they may also limit individual disclosure when participants feel constrained by group dynamics. However, research evidence suggests that self-disclosure raises feelings of empathy in others and consequently increases their level of self-disclosure. Nevertheless, the extent to which the cultural context of the RC and Germany influences the phenomenon is unknown.

In more detail, although the focus of the study is mainly on educators' professional experiences rather than personal morally and psycho-emotionally traumatic events, discussing students' or even personal moral distress may evoke emotional discomfort for some participants. Thus, participants will be informed that they may decline to answer any question or withdraw from the discussion at any time without consequence. The moderator will remain attentive to signs of discomfort and will pause or redirect the discussion if necessary.

In addition, research data show that individuals who experience severe symptoms of mental and physical disorders, including symptoms of secondary trauma or low levels of general health, most often do not participate in studies. Therefore, the proportion of individuals experiencing these symptoms as well as the description of their intensity are expected to be underestimated.

Second, the study will be conducted within two educational and clinical contexts (Greek-Cypriot and German), which may limit the generalizability of the findings to other cultural, institutional, or healthcare systems. Nevertheless, the inclusion of multiple contexts is expected to enhance the transferability of insights by enabling comparisons across settings [30],[32]. Moreover, as with all qualitative research, the interpretation of findings may be influenced by the researchers' perspectives and prior experiences. To enhance analytical rigor, the research team will engage in reflexive discussions during the analysis process and compare interpretations among team members [33]. This collaborative approach aims to reduce the potential impact of individual researcher bias.

Third, as we focus on academics' and educators' perspectives, we do not directly capture the living experiences of midwifery students or clinical mentors. While this perspective is intentional and central to the study objectives, it represents only one dimension of a complex educational and clinical phenomenon. Future research incorporating students' and mentors' perspectives would provide a more comprehensive understanding. Finally, as a protocol study, the anticipated findings and implications are necessarily speculative and dependent on the quality and depth of participant engagement. Despite these limitations, the proposed study design is considered appropriate for generating exploratory, context-sensitive knowledge that can inform subsequent intervention development and larger-scale studies.

## Use of AI tools declaration

AI tools have been used for translation and references management.

## References

[1] Jameton A (2017). What moral distress in nursing history could suggest about the future of health care. AMA J Ethics.

[2] Bennett M (2025). Moral distress, disempowerment, and responsibility. Philos Med.

[3] Morley G, Bradbury-Jones C, Ives J (2019). What is ‘moral distress’ in nursing? A feminist empirical bioethics study. Nursing Ethics.

[4] Oelhafen S, Cignacco E (2018). Moral distress and moral competences in midwifery: A latent variable approach. J Health Psychol.

[5] Kingma E (2021). Harming one to benefit another: the paradox of autonomy and consent in maternity care. Bioethics.

[6] Davison C (2020). Feminism, midwifery and the medicalisation of birth. Br J Midwifery.

[7] Foster W, McKellar L, Fleet JA (2024). The barometer of moral distress in midwifery: a pilot study. Women Birth.

[8] Foster W, McKellar L, Fleet J (2022). Moral distress in midwifery practice: a concept analysis. Nurs Ethics.

[9] Foster W, McKellar L, Fleet JA (2023). Moral distress in midwifery practice: a Delphi study. Women Birth.

[10] Megregian M, Low LK, Emeis C (2021). Essential components of midwifery ethics education: results of a Delphi study. Midwifery.

[11] Rost M, Montagnoli C, Eichinger J (2025). Causes of moral distress among midwives: a scoping review. Nursing Ethics.

[12] Albert JS, Younas A, Sana S (2020). Nursing students' ethical dilemmas regarding patient care: An integrative review. Nursing Ethics.

[13] Heng TJT, Shorey S (2023). Experiences of moral distress in nursing students: a qualitative systematic review. Nurse Educ Today.

[14] Apay SE, Gürol A, Gür EY (2020). Midwifery students' reactions to ethical dilemmas encountered in outpatient clinics. Nursing Ethics.

[15] Deschenes S, van Kessel C (2024). Moral distress and nursing education: curricular and pedagogical strategies for a complex phenomenon. Health Care Anal.

[16] Basanta MF, Bouzas-González M, Coronado C (2022). Moral experiences in caring for voluntary pregnancy losses: A meta-ethnography. Nursing Ethics.

[17] Megregian M, Low LK, Emeis C (2016). Midwives' experiences of ethics education and ethical dilemmas: A qualitative study. Nursing Ethics.

[18] Megregian M, Kane Low L, Emeis C (2020). “I'm sure we talked about it”: midwives' experiences of ethics education and ethical dilemmas—a qualitative study. Women Birth.

[19] Megregian M, Low LK, Emeis C (2021). Midwifery students' expectations of and experiences with ethics education: a qualitative study. Nurse Educ Today.

[20] Mpeli MR (2018). Analysis of self-evaluated ethical competence of midwifery students at a selected nursing college in the Free State. Curationis.

[21] Kabirian M, Gooshki ES, Khadivzadeh T (2024). The process of professional ethics development in midwifery students: a grounded theory study. Iran J Nurs Midwifery Res.

[22] Coldridge L, Davies S (2017). “Am I too emotional for this job?” An exploration of student midwives' experiences of coping with traumatic events in the labour ward. Midwifery.

[23] Foster W, McKellar L, Fleet JA (2022). Exploring moral distress in Australian midwifery practice. Women Birth.

[24] Moghadam MH, Nabavi FH, Miri HH (2024). Participatory management effects on nurses' organizational support and moral distress. Nurs Ethics.

[25] Christodoulou-Fella M, Papathanassoglou E, Middleton N (2018). Qualitative investigation of moral distress in Cypriot mental health nurses: preliminary results. Nurs Care Res.

[26] Honkavuo L (2022). Midwifery students' experiences of support for ethical competence: a phenomenological study. Nurs Ethics.

[27] Jefford E, Nolan S, Jomeen J (2022). Giving midwives a voice: qualitative perspectives of an empowering decision-making workshop. J Clin Nurs.

[28] Tong A, Peter Sainsbury P, Craig J (2007). Consolidated criteria for reporting qualitative research (COREQ): a 32-item checklist for interviews and focus groups. Int J Qual Health Care.

[29] Karanikola MNK (2019). Content analysis in critical and emergency care: a discussion paper. CONNECT World Crit Care Nurs.

[30] Polit DF, Beck CT (2021). Essentials of Nursing Research: Appraising Evidence for Nursing Practice.

[31] Guest G, Namey E, Taylor J (2017). Comparing focus groups and individual interviews: Findings from a randomized study. Int J Soc Res Methodol.

[32] Moraiti A, Papadatou D (2010). The use of focused discussion groups in collecting qualitative data. Nosileftiki.

[33] Munhall PL (2002). Nursing Research: A Qualitative Perspective.

[34] Buchbinder M, Browne A, Berlinger N (2024). Moral stress and moral distress: Confronting challenges in healthcare systems under pressure. Am J Bioeth.

[35] Mewborn EK, Fingerhood ML, Johanson L (2023). Examining moral injury in clinical practice: A narrative literature review. Nurs Ethics.

[36] VanderWeele TJ, Wortham JS, Carey LB (2025). Moral trauma, moral distress, moral injury, and moral injury disorder: definitions and assessments. Front Psychol.

[37] Jain A, Torres LD, Teoh K (2022). The impact of national legislation on psychosocial risks on organisational action plans, psychosocial working conditions, and employee work-related stress in Europe. Soc Sci Med.

[38] Figley CR (1995). Compassion fatigue: coping with secondary traumatic stress disorder in those who treat the traumatized.

[39] Sadock JB, Alcott Sadock V, Ruiz P (2017). Kaplan & Sadock's Comprehensive Textbook of Psychiatry.

[40] Leinweber J, Creedy DK, Rowe H (2017). Responses to birth trauma and prevalence of posttraumatic stress among Australian midwives. Women Birth.

[41] Yılmaz S, Ordu Y, Aktaş D (2025). Trauma level of midwifery students after the catastrophe of the century Turkey earthquake: a cross-sectional descriptive study. Disaster Med Public Health Prep.

[42] Christodoulou-Fella M, Middleton N, Papathanassoglou ED (2017). Exploration of the association between nurses' moral distress and secondary traumatic stress syndrome: implications for patient safety in mental health services. Biomed Res Int.

[43] Gamvrouli M, Karanikola M, Triantafyllou C (2025). Levels of moral distress, secondary traumatic stress, general health, and empathy among nursing staff in eight public hospitals in Greece: a cross-sectional study. Worldviews Evid Based Nurs.

[44] Moisoglou I, Katsiroumpa A, Fradelos EC (2025). Association between nursing work environment, burnout, and turnover intention: a cross-sectional study in Greece. AIMS Public Health.

[45] Small K, Warton C, Fenwick J (2025). The psychological impact of working as a midwife in Australia: findings from a scoping review. Midwifery.

[46] Dehkordi LM, Kianian T, Nasrabadi AN (2024). Nursing students' experience of moral distress in clinical settings: a phenomenological study. Nurs Open.

[47] Doody O, Slevin E, Taggart L (2013). Focus group interviews in nursing research: part 1. Br J Nurs.

